# Nicotinamide Mononucleotide Protects against Retinal Dysfunction in a Murine Model of Carotid Artery Occlusion

**DOI:** 10.3390/ijms232314711

**Published:** 2022-11-25

**Authors:** Deokho Lee, Yohei Tomita, Yukihiro Miwa, Heonuk Jeong, Ari Shinojima, Norimitsu Ban, Shintaro Yamaguchi, Ken Nishioka, Kazuno Negishi, Jun Yoshino, Toshihide Kurihara

**Affiliations:** 1 Laboratory of Photobiology, Keio University School of Medicine, Tokyo 160-8582, Japan; 2 Department of Ophthalmology, Keio University School of Medicine, Tokyo 160-8582, Japan; 3 Aichi Animal Eye Clinic, Nagoya 466-0827, Japan; 4 Department of Internal Medicine, Keio University School of Medicine, Tokyo 160-8582, Japan

**Keywords:** nicotinamide mononucleotide, oxidative stress, common carotid artery occlusion, retinal ischemia, neuroprotection

## Abstract

Cardiovascular abnormality-mediated retinal ischemia causes severe visual impairment. Retinal ischemia is involved in enormous pathological processes including oxidative stress, reactive gliosis, and retinal functional deficits. Thus, maintaining retinal function by modulating those pathological processes may prevent or protect against vision loss. Over the decades, nicotinamide mononucleotide (NMN), a crucial nicotinamide adenine dinucleotide (NAD^+^) intermediate, has been nominated as a promising therapeutic target in retinal diseases. Nonetheless, a protective effect of NMN has not been examined in cardiovascular diseases-induced retinal ischemia. In our study, we aimed to investigate its promising effect of NMN in the ischemic retina of a murine model of carotid artery occlusion. After surgical unilateral common carotid artery occlusion (UCCAO) in adult male C57BL/6 mice, NMN (500 mg/kg/day) was intraperitoneally injected to mice every day until the end of experiments. Electroretinography and biomolecular assays were utilized to measure ocular functional and further molecular alterations in the retina. We found that UCCAO-induced retinal dysfunction was suppressed, pathological gliosis was reduced, retinal NAD^+^ levels were preserved, and the expression of an antioxidant molecule (nuclear factor erythroid-2-related factor 2; *Nrf2*) was upregulated by consecutive administration of NMN. Our present outcomes first suggest a promising NMN therapy for the suppression of cardiovascular diseases-mediated retinal ischemic dysfunction.

## 1. Introduction

Cardiovascular dysfunction-induced retinal injury is a type of the severe vision-threatening diseases. As blood (including oxygen) to the retina is directly supplied through the ophthalmic artery stretched from the internal carotid artery (a branch of the common carotid artery), the retina is highly susceptible to cardiovascular dysfunction [1]. When cardiovascular abnormality (including carotid artery stenosis or occlusion) occurs, retinal ischemia can be evoked in human and murine [2,3,4], finally leading to transient or permanent visual impairment.

Retinal ischemia contains complicated pathological processes including oxidative stress, reactive gliosis, and retinal functional deficits [5,6]. As effective treatment has not yet been developed for retinal ischemia-induced retinal dysfunction, studies on searching for promising therapeutics to protect against retinal ischemia-induced retinal dysfunction have been attempted at the preclinical stage [6].

Nicotinamide mononucleotide (NMN) is an important precursor of nicotinamide adenine dinucleotide (NAD^+^), an essential molecule required for various cellular functions [7]. Maintaining NAD^+^ biosynthesis has been considered important for the prevention of a variety of age-related metabolic diseases and disorders, including obesity, insulin resistance, and diabetes [8,9,10]. Previously, NMN was also reported to prevent or protect against retinal damages (the inner and/or outer retina) in light-induced retinopathy, retinal detachment, or retinal ischemia/reperfusion injury [11,12,13]. It has been speculated that therapeutic effects of NMN might be associated with maintenance of levels of NAD^+^. However, a protective effect of NMN has not yet been examined in cardiovascular disease-mediated retinal dysfunction.

Therefore, in the current study, we first investigated whether NMN treatment could show a promising protective effect in the ischemic retina of a murine model of carotid artery occlusion.

## 2. Results

### 2.1. Consecutive Treatment of NMN Protects against Retinal Dysfunction in a Mouse Model of Unilateral Common Carotid Artery Occlusion

**Figure 1:**
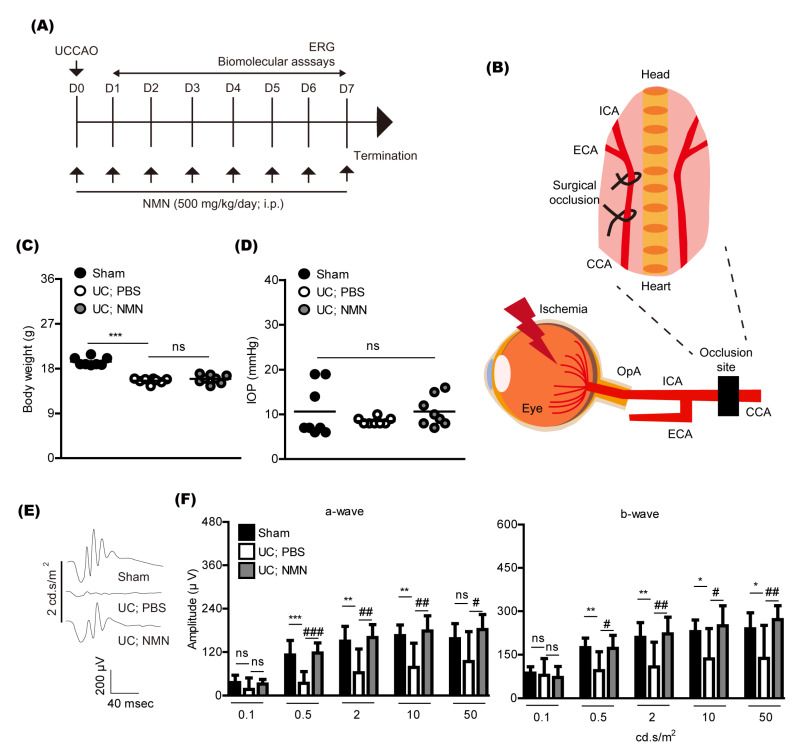
Retinal functional protection by continuous NMN treatment. (**A**) A general experimental scheme. UCCAO, unilateral common carotid artery occlusion; D, day; ERG, electroretinography; i.p., intraperitoneal injection; NMN, nicotinamide mononucleotide. (**B**) An anatomical view on the induction of retinal ischemia by surgical UCCAO. Surgical permanent occlusion of the common carotid artery (CCA) could induce occlusion of the internal carotid artery (ICA), finally leading to occlusion of the ophthalmic artery (OpA) which supplies the retina. (**C**) Quantitative analyses (n = 8 per group) demonstrated that the body weight was significantly reduced by UCCAO. There was no dramatic difference in the body weight between PBS-treated and NMN-treated UCCAO-operated mice. *** *p* <  0.001; ns, not significant. One-way ANOVA followed by a Bonferroni post hoc test. A graph is shown as mean with actual values (mean and standard deviation, sham: 19.41, 0.83; UC; PBS: 15.60, 0.50; UC; NMN: 15.94, 0.86). (**D**) Quantitative analyses (n = 8 per group) demonstrated that there was no difference in intraocular pressure (IOP) among all group. ns, not significant. One-way ANOVA followed by a Bonferroni post hoc test. A graph is shown as mean with actual values (mean and standard deviation, sham: 10.63, 5.78; UC; PBS: 8.50, 0.75; UC; NMN: 10.63, 3.37). (**E**,**F**) Representative waveforms (2 cd.s/m^2^) of ERG and quantitative analyses (n = 7–12 per group) demonstrated that the ERG amplitudes decreased 7 days after UCCAO. NMN treatment suppressed reductions in the ERG amplitudes, flashed with various standardized intensities (0.1, 0.5, 2, 10, or 50 cd.s/m^2^). * *p* <  0.05, ** *p* <  0.01, *** *p* <  0.001; ^#^ *p* <  0.05, ^##^ *p* <  0.01, ^###^ *p* <  0.001. One-way ANOVA followed by a Bonferroni post hoc test. Graphs are shown as mean  ±  standard deviation. UC, UCCAO.

To examine whether NMN treatment could show a protective effect against retinal dysfunction caused by unilateral common carotid artery occlusion (UCCAO) in adult mice, NMN (500 mg/kg) was intraperitoneally injected to mice after UCCAO (Figure 1A). NMN was continuously provided every day until the end of whole experiments. The concentration of NMN was determined based on our previous paper and other groups’ reports [11,12,13,14], which is general for various experimental murine models. UCCAO was conducted using permanent surgical occlusion in the right common carotid artery (Figure 1B), according to our previous publications [15,16,17]. Retinal ischemia could be evoked as retinal blood is supplied through the ophthalmic artery (OpA) connected with the internal carotid artery (ICA), one of the branches of the common carotid artery (CCA). As eyelid drooping was generally detected as a sign for a successful UCCAO surgery [15,16,17], we grossly examined eyelids of all mice after UCCAO, and found 100% rate of eyelid drooping in UCCAO groups, similar to our previous observations.

As a general parameter, we screened body weight (Figure 1C) and intraocular pressure (Figure 1D) after UCCAO. After UCCAO, the body weight was reduced, which has been consistently seen in the murine UCCAO model [18,19]. There was no change in the body weight by NMN treatment. There was no dramatic difference in intraocular pressure among groups, similar with that observed in the murine UCCAO model.

Previously, we found that retinal dysfunction was seen 7 days after UCCAO [16]. We reproduced this finding in our current system (Figure 1E,F). Continuous NMN treatment significantly suppressed its reductions.

### 2.2. Consecutive Treatment of NMN Reduces Pathological Gliosis in a Mouse Model of Unilateral Common Carotid Artery Occlusion

**Figure 2:**
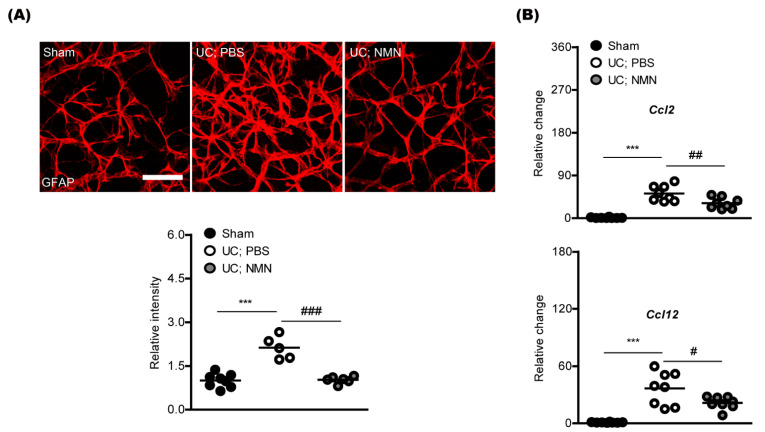
Modulation of pathological retinal gliosis and inflammation by consecutive NMN treatment. (**A**) Representative images and quantitative analyses (n = 5–8 per group) demonstrated that NMN treatment suppressed increases in GFAP immunoreactivities induced by UCCAO. Scale bar, 50 μm. *** *p* <  0.001; ^###^ *p* <  0.001. One-way ANOVA followed by a Bonferroni post hoc test. A graph is shown as mean with actual values (mean and standard deviation, sham: 1.00, 0.23; UC; PBS: 2.12, 0.39; UC; NMN: 1.02, 0.12). (**B**) Quantitative analyses (n = 8 per group) demonstrated that NMN administration reduced upregulation in *Ccl2* and *Ccl12* mRNA expressions in the ischemic retina. *** *p* <  0.001; ^#^ *p* <  0.05, ^##^ *p* <  0.01. One-way ANOVA followed by a Bonferroni post hoc test. Graphs are shown as mean with actual values (mean and standard deviation, sham: 1.00, 1.02; UC; PBS: 51.95, 16.42; UC; NMN: 31.46, 11.72; sham: 1.00, 0.38; UC; PBS: 36.54, 17.33; UC; NMN: 21.50, 6.54). UC, UCCAO.

We examined whether NMN treatment could reduce pathological gliosis caused by UCCAO in mice. Previously, there were increases in retinal glial activation (especially, an increased immunoreactivity in glial fibrillary acidic protein; GFAP) after UCCAO [16]. Consistent with the previous report, we could detect glial activation, analyzed with the GFAP immunoreactivity (Figure 2A). Furthermore, NMN treatment reduced pathological gliosis. Additionally, we screened two inflammatory glial chemokines (*Ccl2* and *Ccl12*) as their expressions have been reported to increase after UCCAO [17,18]. Induction in chemokine ligands *Ccl2* and *Ccl12* mRNA expressions was reduced by NMN treatment under the same condition (Figure 2B).

### 2.3. Consecutive Treatment of NMN Preserves Redox Balance and Activates an Antioxidant Pathway in a Mouse Model of Unilateral Common Carotid Artery Occlusion

**Figure 3:**
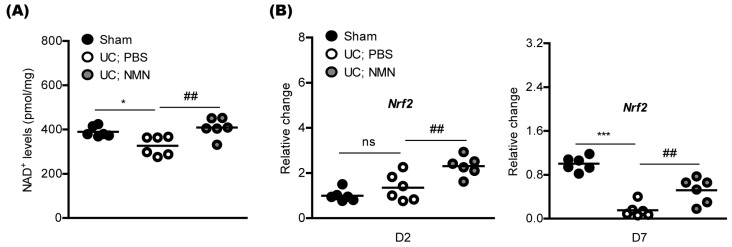
Modulation of redox balance and an antioxidant pathway by NMN treatment. (**A**) Quantitative analyses (n = 6 per group) demonstrated that retinal NAD^+^ levels were significantly reduced by UCCAO. NMN treatment significantly suppressed its reduction. * *p* <  0.05; ^##^ *p* <  0.01. One-way ANOVA followed by a Bonferroni post hoc test. A graph is shown as mean with actual values (mean and standard deviation, sham: 390.0, 23.38; UC; PBS: 326.2, 43.12; UC; NMN: 408.7, 44.13). (**B**) Quantitative analyses (n = 6 per group) demonstrated that NMN administration increased *Nrf2* mRNA expression in the ischemic retina. *** *p* <  0.001; ^##^ *p* <  0.01; ns, not significant. One-way ANOVA followed by a Bonferroni post hoc test. A graph is shown as mean with actual values (mean and standard deviation, sham: 1.00, 0.26; UC; PBS: 1.35, 0.60; UC; NMN: 2.30, 0.43; sham: 1.00, 0.12; UC; PBS: 0.15, 0.12; UC; NMN: 0.51, 0.23). UC, UCCAO; D, day.

We examined whether NMN treatment could preserve redox balance and activate an antioxidant pathway. Numerous previous reports demonstrated that supplementation with NMN could increase NAD^+^ biosynthesis in various cell types [20,21,22]. We applied this concept to our current system, and found the preservation of NAD^+^ levels in the UCCAO-induced ischemic retina by continuous NMN treatment (Figure 3A).

Next, as NMN treatment has also been reported to increase antioxidant genes (especially, nuclear factor erythroid-2-related factor 2; *Nrf2*) in various cell types in vitro and in vivo [12,23,24,25,26]. Therefore, we examined whether NMN treatment could increase *Nrf2* mRNA expression under our current condition (Figure 3B). On day 2 after UCCAO, we found that *Nrf2* mRNA expression increased by NMN treatment, while there was no significant difference in its expression between sham and UCCAO groups (Figure 3B). On day 7 after UCCAO, we found decreases in its expression, while NMN treatment dramatically suppressed the reductions.

### 2.4. Consecutive Treatment of NMN Does Not Affect Retinal Thickness in a Mouse Model of Unilateral Common Carotid Artery Occlusion

**Figure 4:**
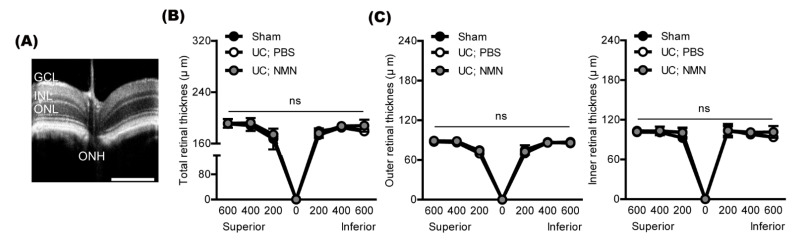
Screening of alterations in retinal thickness by NMN treatment. (**A**) A representative retinal picture of the optical coherence tomography (OCT). GCL, the ganglion cell layer; INL, the inner nuclear layer; ONL, the outer nuclear layer; ONH, the optic nerve head. Scale bar, 500 μm. (**B**,**C**) Quantitative analyses showed that NMN treatment did not change retinal thickness in UCCAO-operated mice, analyzed at 200, 400, and 600 μm from ONH (n = 5–6 per group). ns, not significant. Two-way ANOVA followed by a Bonferroni post hoc test. Graphs are shown as mean  ±  standard deviation. UC, UCCAO.

According to our previous UCCAO papers, retinal thickness was not dramatically altered by UCCAO in mice. Nonetheless, we examined whether NMN treatment may affect retinal thickness in UCCAO-operated mice (Figure 4). There was no dramatic difference in retinal thickness (total, outer, and inner) among all groups. Based on our current functional and histological outcomes, NMN treatment may work on retinal functional protection without affecting retinal thickness in UCCAO-operated mice.

## 3. Discussion

The present study demonstrated that retinal functional impairment and pathological retinal gliosis could be induced by cardiovascular dysfunction, and continuous NMN treatment could show therapeutic effects against such injuries. Furthermore, redox imbalance under the retinal ischemic condition was modulated by continuous NMN treatment. Although promising NMN therapy in not only age-dependent or systemic metabolism-mediated diseases and disorders but also several retinopathies has been recently suggested [8,9,10], as far as we know, our current report is the first to expand the protective role of NMN in a murine model of permanent surgical occlusion of the unilateral common carotid artery.

We found that UCCAO-induced retinal dysfunction and pathological gliosis were reduced by consecutive NMN treatment. Similar aspects were described in previous studies from ours and others. We recently found that retinal ischemia/reperfusion injury-induced retinal dysfunction was lessened by consecutive NMN treatment [12]. Chen et al. showed that NMN treatment reduced cell death in the outer layer at the acute stage of retinal detachment [11]. Furthermore, NMN treatment suppressed pathological gliosis at the late stage of retinal detachment. Lin et al. showed that NMN treatment reduced retinal dysfunction in mice lacking *Nampt* and in mice of light-induced retinal damages [13]. In this aspect, NMN therapy might be promising for prevention or protection of various retinal diseases.

NMN is one of the natural compounds to boost NAD^+^ biosynthesis. In mammals, NMN can be produced from nicotinamide (NAM, a form of vitamin B3 found in various foods) by nicotinamide phosphoribosyltransferase (NAMPT), the rate-limiting enzyme [21,27,28]. NMN can also be synthesized from nicotinamide riboside (NR) through NR kinase-mediated phosphorylation reaction. NMN can be converted into NAD^+^ by NMN adenylyltransferase (NMNAT). In this regard, NMN supplementation could increase NAD^+^ biosynthesis. In fact, numerous experimental studies showed its relationship under their experimental conditions in not only the central nervous system but also other types of body systems [21,27,28]. Chen et al. showed that retinal NAD^+^ levels increased by NMN treatment in the retinal detachment-induced damaged retina [11]. In our current study, we found that retinal NAD^+^ levels also increased by NMN treatment in the UCCAO-operated ischemic retina. Taken together, its event might be highly conserved. Nonetheless, more studies are needed on which strategies (administration methods, concentrations, or durations for NMN treatment) could effectively activate this biosynthesis.

Therapeutic roles of NMN are generally explained with upregulation of antioxidants to protect cells against redox imbalance. Based on our and other previous reports, the antioxidant function of NMN has been associated with the *Nrf2* antioxidant pathway [12,23,24,25,26]. We recently demonstrated that NMN treatment increased *Nrf2* expression and cellular protective properties in retinal 661W cells under CoCl_2_-induced pseudohypoxic oxidative stress conditions [12]. Pu et al. demonstrated that NMN treatment showed protective effects against high glucose-evoked cellular damages in human corneal epithelial cells via the *Nrf2* signaling pathway [25]. Wei et al. demonstrated that NMN treatment attenuated intracerebral hemorrhage-induced cellular damages in the central nervous system through the *Nrf2* signaling pathway [26]. In our current data, NMN treatment constantly increased *Nrf2* expression in the ischemic retina under the UCCAO-induced redox imbalanced condition. Taken together, our present outcome also supports the notion that the therapeutic role of NMN might be associated with the *Nrf2* antioxidant signaling pathway in various eukaryotic cells and tissues.

In the murine model of UCCAO itself, there was no dramatic change in retinal thickness in our current experimental system. Its finding has been consistently detected in our previous publications [15,16,17]. Nonetheless, retinal thickness could be affected by retinal ischemic insults in humans [29,30,31,32]. Although there exist gaps between mice and humans, it can be speculated that retinal ischemia in humans is closely combined with systemic metabolic stress [33,34,35], while the experimental UCCAO murine model has no such stress. To solve this issue, the UCCAO model can be combined with systemic metabolic dysregulation such as streptozotocin injection (to induce systemic diabetic conditions) or genetic manipulation (to cause metabolic diseases or conditions of interest) [36,37,38,39]. With this novel model of UCCAO, more clinically relevant findings could be obtained with NMN therapy, which will be further studied.

In summary, we firstly attempted to apply the currently promising NMN therapy to a murine model of unilateral common carotid artery occlusion. As the outcomes, retinal functional damages and pathological gliosis were suppressed by consecutive NMN treatment. Furthermore, retinal redox balance was preserved by consecutive NMN treatment along with upregulating the antioxidant pathway. Although further understandings on the mechanism of action of NMN therapy in retinal ischemia are desired, we suggest that NMN supplements can be one of the promising therapeutic strategies for protecting against ischemic retinopathy based on our brief observations.

## 4. Materials and Methods

### 4.1. Animal, Unilateral Common Carotid Artery Occlusion, and NMN Treatment

Whole animal experimental procedures were followed by the Ethics Committee on Animal Research of Keio University School of Medicine (#16017), the International Standards of Animal Care and Use, Animal Research: Reporting in Vivo Experiments, and the ARVO Statement for the Use of Animals in Ophthalmic and Vision Research.

Adult black male mice (C57BL/6, 5 weeks old) were obtained from CLEA Japan (Tokyo, Japan). After randomizing mice, we performed the UCCAO surgery, as previously described [15,16,17]. Briefly, the neck areas of mice were open to observe the right carotid arteries. The right CCA was tightly occluded using 6–0 silk sutures. After wound suturing, mice were recovered with warm pads to maintain body temperature. When mice were awake, they were back to cages for the further experiments. After mice were fully recovered (around 4 h after the surgery), intraperitoneal NMN (500 mg/kg) injection was consistently performed every day for 24 h interval.

### 4.2. Electroretinography (ERG) and Optical Coherence Tomography (OCT)

We performed ERG, as previously indicated in our papers [15,16]. Briefly, after dark adaptation for more than 12 h, mice were anesthetized using a mixture of medetomidine (7.5 μg/100 μL; Orion, Espoo, Finland), midazolam (40 μg/100 μL; Sandoz, Tokyo, Japan), and butorphanol tartrate (50 μg/100 μL; Meiji Seika Pharma, Tokyo, Japan) [40]. Scotopic ERG responses were recorded using a PuREC ERG acquisition system (MAYO, Inazawa, Japan). Various standardized light stimuli were evoked to measure the amplitudes of a-wave and b-wave.

Envisu R4310 OCT (Leica, Wetzlar, Germany) was performed as previously indicated in our papers [15,16]. Briefly, after anesthesia, mice were immediately placed on an OCT platform. After adjustment for positioning the retina to the OCT camera, retinal images at 200, 400, and 600 μm from the optic nerve head were captured. Retinal thickness was measured using the software provided by Envisu R4310 OCT.

### 4.3. Immunohistochemistry (IHC)

We performed IHC, as previously described in our papers [15,16,17]. After making flat-mounted retinas from the eyes, a primary antibody (GFAP 1:400, Cat #13-0300, Thermo Fisher Scientific, Waltham, MA, USA) was added to the retinas. After overnight incubation, the retinas were washed with PBS including 0.1% Triton three times. Species-appropriate fluorescence-conjugated secondary antibodies (Thermo Fisher Scientific, Waltham, MA, USA) were added to the retinas for 2 h at room temperature. After generally washing with PBS including 0.1% Triton three times, the retinas were mounted with cover slips and examined using the LSM710 microscope (Carl Zeiss, Jena, Germany).

### 4.4. Quantitative PCR (qPCR)

Whole procedures for qPCR analysis were as same as described in our previous publications [15,16,17]. A series of RNA-cDNA-qPCR kits (Qiagen, Velno, Netherlands; TOYOBO, Osaka, Japan; Applied Biosystems, Waltham, MA, USA) were utilized to prepare RNA samples, synthesize cDNA, and conduct qPCR. The primer sequence used in the present study is entered in Table 1. The generally used ΔΔCT analysis protocol was utilized to determine relative fold changes.

### 4.5. Nicotinamide Adenine Dinucleotide (NAD^+^) Assay

We performed NAD^+^ assay using a commercially available kit by following manufacturer’s instructions (Cat #ab65348, Abcam, San Francisco, CA, USA). Briefly, fresh retinal samples (within 1 h after the last NMN treatment) were homogenized in extraction buffer in the kit. Then, concentrations of total NAD and decomposed NAD^+^ were measured using the extracted samples by a plate reader (Synergy HT Multi-Mode, Winooski, VT, USA). NAD^+^ was calculated with the equation NAD^+^ = total NAD − decomposed NAD^+^.

### 4.6. Statistical Analysis

Statistical significance for all experimental values was determined using one or two-way ANOVA followed by a Bonferroni post hoc test depending on the data set. Statistical significance was regarded when *p* < 0.05.

## Figures and Tables

**Table 1:** Primer list.

Name	Direction	Sequence (5’ → 3’)	Accession Number
*Hprt*	Forward	TCAGTCAACGGGGGACATAAA	NM_013556.2
	Reverse	GGGGCTGTACTGCTTAACCAG	
*Nrf2*	Forward	TAGATGACCATGAGTCGCTTGC	NM_010902.4
	Reverse	GCCAAACTTGCTCCATGTCC	
*Ccl2*	Forward	CCCAATGAGTAGGCTGGAGA	NM_011333.3
	Reverse	TCTGGACCCATTCCTTCTTG	
*Ccl12*	Forward	GCTACAGGAGAATCACAAGCAGC	NM_011331.3
	Reverse	ACGTCTTATCCAAGTGGTTTATGG	

## Data Availability

The current study’s data are available on request from the corresponding author.

## References

[1] Vilares-Morgado R., Nunes H.M.M., Dos Reis R.S., Barbosa-Breda J. (2022). Management of ocular arterial ischemic diseases: A review. Graefe’s Arch. Clin. Exp..

[2] Long C.P., Chan A.X., Bakhoum C.Y., Toomey C.B., Madala S., Garg A.K., Freeman W.R., Goldbaum M.H., DeMaria A.N., Bakhoum M.F. (2021). Prevalence of subclinical retinal ischemia in patients with cardiovascular disease-a hypothesis driven study. EClinicalMedicine.

[3] Lee D., Tomita Y., Yang L., Negishi K., Kurihara T. (2022). Ocular Ischemic Syndrome and Its Related Experimental Models. Int. J. Mol. Sci..

[4] Vestergaard N., Cehofski L.J., Honoré B., Aasbjerg K., Vorum H. (2019). Animal Models Used to Simulate Retinal Artery Occlusion: A Comprehensive Review. Transl. Vis. Sci. Technol..

[5] Osborne N.N., Casson R.J., Wood J.P., Chidlow G., Graham M., Melena J. (2004). Retinal ischemia: Mechanisms of damage and potential therapeutic strategies. Prog. Retin. Eye Res..

[6] Minhas G., Morishita R., Anand A. (2012). Preclinical models to investigate retinal ischemia: Advances and drawbacks. Front. Neurol..

[7] Nadeeshani H., Li J., Ying T., Zhang B., Lu J. (2022). Nicotinamide mononucleotide (NMN) as an anti-aging health product-Promises and safety concerns. J. Adv. Res..

[8] Okabe K., Yaku K., Tobe K., Nakagawa T. (2019). Implications of altered NAD metabolism in metabolic disorders. J. Biomed. Sci..

[9] Yoshino J., Mills K.F., Yoon M.J., Imai S. (2011). Nicotinamide mononucleotide, a key NAD(+) intermediate, treats the pathophysiology of diet- and age-induced diabetes in mice. Cell Metab..

[10] Hong W., Mo F., Zhang Z., Huang M., Wei X. (2020). Nicotinamide Mononucleotide: A Promising Molecule for Therapy of Diverse Diseases by Targeting NAD+ Metabolism. Front. Cell Dev. Biol..

[11] Chen X., Amorim J.A., Moustafa G.A., Lee J.J., Yu Z., Ishihara K., Iesato Y., Barbisan P., Ueta T., Togka K.A. (2020). Neuroprotective effects and mechanisms of action of nicotinamide mononucleotide (NMN) in a photoreceptor degenerative model of retinal detachment. Aging.

[12] Lee D., Tomita Y., Miwa Y., Shinojima A., Ban N., Yamaguchi S., Nishioka K., Negishi K., Yoshino J., Kurihara T. (2022). Nicotinamide Mononucleotide Prevents Retinal Dysfunction in a Mouse Model of Retinal Ischemia/Reperfusion Injury. Int. J. Mol. Sci..

[13] Lin J.B., Kubota S., Ban N., Yoshida M., Santeford A., Sene A., Nakamura R., Zapata N., Kubota M., Tsubota K. (2016). NAMPT-Mediated NAD(+) Biosynthesis Is Essential for Vision In Mice. Cell Rep..

[14] Mills K.F., Yoshida S., Stein L.R., Grozio A., Kubota S., Sasaki Y., Redpath P., Migaud M.E., Apte R.S., Uchida K. (2016). Long-Term Administration of Nicotinamide Mononucleotide Mitigates Age-Associated Physiological Decline in Mice. Cell Metab..

[15] Lee D., Nakai A., Miwa Y., Tomita Y., Serizawa N., Katada Y., Hatanaka Y., Tsubota K., Negishi K., Kurihara T. (2021). Retinal Degeneration in a Murine Model of Retinal Ischemia by Unilateral Common Carotid Artery Occlusion. BioMed Res. Int..

[16] Lee D., Jeong H., Miwa Y., Shinojima A., Katada Y., Tsubota K., Kurihara T. (2021). Retinal dysfunction induced in a mouse model of unilateral common carotid artery occlusion. PeerJ.

[17] Lee D., Kang H., Yoon K.Y., Chang Y.Y., Song H.B. (2020). A mouse model of retinal hypoperfusion injury induced by unilateral common carotid artery occlusion. Exp. Eye Res..

[18] Lee D., Tomita Y., Miwa Y., Jeong H., Mori K., Tsubota K., Kurihara T. (2021). Fenofibrate Protects against Retinal Dysfunction in a Murine Model of Common Carotid Artery Occlusion-Induced Ocular Ischemia. Pharmaceuticals.

[19] Lee D., Tomita Y., Jeong H., Miwa Y., Tsubota K., Negishi K., Kurihara T. (2021). Pemafibrate Prevents Retinal Dysfunction in a Mouse Model of Unilateral Common Carotid Artery Occlusion. Int. J. Mol. Sci..

[20] Shade C. (2020). The Science Behind NMN-A Stable, Reliable NAD+Activator and Anti-Aging Molecule. Integr. Med..

[21] Yoshino J., Baur J.A., Imai S.I. (2018). NAD(+) Intermediates: The Biology and Therapeutic Potential of NMN and NR. Cell Metab..

[22] Nagahisa T., Yamaguchi S., Kosugi S., Homma K., Miyashita K., Irie J., Yoshino J., Itoh H. (2022). Intestinal Epithelial NAD+ Biosynthesis Regulates GLP-1 Production and Postprandial Glucose Metabolism in Mice. Endocrinology.

[23] Liu X., Dilxat T., Shi Q., Qiu T., Lin J. (2022). The combination of nicotinamide mononucleotide and lycopene prevents cognitive impairment and attenuates oxidative damage in D-galactose induced aging models via Keap1-Nrf2 signaling. Gene.

[24] Luo C., Ding W., Yang C., Zhang W., Liu X., Deng H. (2022). Nicotinamide Mononucleotide Administration Restores Redox Homeostasis via the Sirt3-Nrf2 Axis and Protects Aged Mice from Oxidative Stress-Induced Liver Injury. J. Proteome Res..

[25] Pu Q., Guo X.X., Hu J.J., Li A.L., Li G.G., Li X.Y. (2022). Nicotinamide mononucleotide increases cell viability and restores tight junctions in high-glucose-treated human corneal epithelial cells via the SIRT1/Nrf2/HO-1 pathway. Biomed. Pharmacother..

[26] Wei C.C., Kong Y.Y., Li G.Q., Guan Y.F., Wang P., Miao C.Y. (2017). Nicotinamide mononucleotide attenuates brain injury after intracerebral hemorrhage by activating Nrf2/HO-1 signaling pathway. Sci. Rep..

[27] She J., Sheng R., Qin Z.H. (2021). Pharmacology and Potential Implications of Nicotinamide Adenine Dinucleotide Precursors. Aging Dis..

[28] Palmer R.D., Elnashar M.M., Vaccarezza M. (2021). Precursor comparisons for the upregulation of nicotinamide adenine dinucleotide. Novel approaches for better aging. Aging Med..

[29] Shariati M.A., Park J.H., Liao Y.J. (2015). Optical coherence tomography study of retinal changes in normal aging and after ischemia. Investig. Ophthalmol. Vis. Sci..

[30] Ho J.K., Stanford M.P., Shariati M.A., Dalal R., Liao Y.J. (2013). Optical coherence tomography study of experimental anterior ischemic optic neuropathy and histologic confirmation. Investig. Ophthalmol. Vis. Sci..

[31] Ackermann P., Brachert M., Albrecht P., Ringelstein M., Finis D., Geerling G., Aktas O., Guthoff R. (2017). Alterations of the outer retina in non-arteritic anterior ischaemic optic neuropathy detected using spectral-domain optical coherence tomography. Clin. Exp. Ophthalmol..

[32] Kwon D.H., Kim Y.C., Kang K.T. (2022). Clinical Significance of Choroidal Thickness in Eyes with Ocular Ischemic Syndrome. Korean J. Ophthalmol. KJO.

[33] Jeong A., Yao X., van Hemert J., Sagong M. (2022). Clinical significance of metabolic quantification for retinal nonperfusion in diabetic retinopathy. Sci. Rep..

[34] Singh C. (2022). Metabolism and Vascular Retinopathies: Current Perspectives and Future Directions. Diagnostics.

[35] Duh E.J., Sun J.K., Stitt A.W. (2017). Diabetic retinopathy: Current understanding, mechanisms, and treatment strategies. JCI Insight.

[36] Tesch G.H., Allen T.J. (2007). Rodent models of streptozotocin-induced diabetic nephropathy. Nephrology.

[37] Rochlani Y., Pothineni N.V., Kovelamudi S., Mehta J.L. (2017). Metabolic syndrome: Pathophysiology, management, and modulation by natural compounds. Ther. Adv. Cardiovasc. Dis..

[38] Barroso I., McCarthy M.I. (2019). The Genetic Basis of Metabolic Disease. Cell.

[39] Yilmaz B.S., Gurung S., Perocheau D., Counsell J., Baruteau J. (2020). Gene therapy for inherited metabolic diseases. J. Mother Child.

[40] Miwa Y., Tsubota K., Kurihara T. (2019). Effect of midazolam, medetomidine, and butorphanol tartrate combination anesthetic on electroretinograms of mice. Mol. Vis..

